# Racial and neighborhood disparities in mortality among hospitalized COVID-19 patients in the United States: An analysis of the CDC case surveillance database

**DOI:** 10.1371/journal.pgph.0000701

**Published:** 2022-11-16

**Authors:** Atarere Joseph, Tarsicio Uribe-Leitz, Tanujit Dey, Joaquim Havens, Zara Cooper, Nakul Raykar

**Affiliations:** 1 Department of Biostatistics and Epidemiology, Harvard T.H. Chan School of Public Health, Boston, Massachusetts, United States of America; 2 Department of Internal Medicine, MedStar Union Memorial Hospital, Baltimore, Maryland, United States of America; 3 Center for Surgery and Public Health, Brigham and Women’s Hospital, Boston, Massachusetts, United States of America; 4 Program in Global Surgery and Social Change, Harvard Medical School, Boston Massachusetts, United States of America; 5 Division of Sport and Health Sciences, Department of Epidemiology, Technical University of Munich, Munich, Germany; 6 Division of Trauma, Burns, and Surgical Critical Care, Department of Surgery, Brigham and Women’s Hospital, Boston, Massachusetts, United States of America; Aarhus University: Aarhus Universitet, DENMARK

## Abstract

**Background:**

Black and Hispanic populations have higher overall COVID-19 infection and mortality odds compared to Whites. Some state-wide studies conducted in the early months of the pandemic found no in-hospital racial disparities in mortality.

**Methods:**

We performed chi-square and logistic regression analyses on the CDC COVID-19 Case Surveillance Restricted Database. The primary outcome of the study was all-cause in-hospital mortality. The primary exposures were racial group (White, Black, Hispanic and Others) and neighborhood type (low vulnerability, moderate vulnerability, high vulnerability, very high vulnerability).

**Findings:**

The overall unadjusted mortality rate was 33% and was lowest among Hispanics. In the fully adjusted models, Blacks and Hispanics had higher overall odds of dying [OR of 1.20 (95% CI 1.15, 1.25) and 1.23 (95% CI 1.17, 1.28) respectively] compared with White patients, and patients from neighborhoods with very high vulnerability had the highest mortality odds in the Northeast, Midwest and overall [Adjusted OR 2.08 (95% CI 1.91, 2.26)]. In the Midwest, Blacks and Hispanics had higher odds of mortality compared with Whites, but this was not observed in other regions.

**Interpretation:**

Among hospitalized COVID-19 patients, Blacks and Hispanics were more likely to die compared to Whites in the Midwest. Patients from highly vulnerable neighborhoods also had the highest likelihood of death in the Northeast and Midwest. These results raise important questions on our efforts to curb healthcare disparities and structural racism in the healthcare setting.

## Introduction

The Coronavirus disease (COVID-19) pandemic, caused by SARS-COV-2 has resulted in millions of deaths worldwide, with more than 1 million of these deaths occurring in the U.S. [1, 2]. Race and neighborhood of residence have been identified as important risk factors for infection and mortality from COVID-19 [3, 4]. Several studies have also shown that Blacks and Hispanics are more likely to be infected with COVID-19 and have a higher burden of mortality than Whites [5–9]. During the early months of the pandemic, residents of neighborhoods in New York with large proportions of Blacks/African Americans were found to be at a significantly higher risk of COVID-19 infection compared to residents of predominantly White neighborhoods [6]. Similarly, the Bronx which has the highest proportion of racial/ethnic minorities, poverty levels and the lowest educational levels had the highest rates of hospitalization and death from COVID-19 in New York [10].

However, some studies conducted among hospitalized COVID-19 patients have found no difference in in-hospital mortality by race [11–13]. A retrospective cohort study conducted on members of an integrated-delivery health system in Louisiana found no racial difference in the hazard of death among hospitalized COVID-19 patients despite a higher overall COVID-19 mortality rate among Blacks [11]. A similar study conducted in New York found that among hospitalized patients, Blacks had lower odds of severe illness and death when compared with Whites [12]. A study conducted among patients admitted to 92 hospitals in 12 U.S. states by Yehia et al. also found no difference in all-cause in-hospital mortality by race [13]. These findings, though, are in contradiction to published reports in other inpatient populations that consistently demonstrate a link between in-hospital mortality and race [14, 15].

The studies in New York and Louisiana, however, were state-level analyses using state and hospital-level datasets [11, 12]. Of the 92 hospitals included in the analysis by Yehia et al., only 2 were from Northeastern states which were the most affected during the early months of the pandemic [13]. Hence, the findings from these studies may not represent the state of disparities in mortality among hospitalized COVID-19 patients nationwide. In addition, although studies in New York have established a relationship between neighborhood racial composition and in-hospital mortality, there are no larger studies evaluating this link on the national level [10]. We sought to characterize in-hospital mortality rates nationwide, determine whether in-hospital mortality for COVID-19 varied based on race and neighborhood type, and evaluate for differences across census regions using a large nationwide database (The CDC COVID-19 Case Surveillance Restricted Access Detailed Data).

## Methods

### Study design

We conducted a cross-sectional study of all hospitalized cases of COVID-19 in the 4 major U.S. census regions between January 1^st^ and November 19^th^, 2020. This study was classified as exempt by the Institutional Review Board (IRB) of Mass General Brigham (MGB) in Boston, Massachusetts. The CDC Case Surveillance database is a de-identified dataset and does not directly involve human subjects as defined by federal regulations and guidance [16].

### Data sources

#### Primary data

The analyses in this study were carried out on the Center for Disease control and prevention (CDC) COVID-19 Case Surveillance Restricted Access Detailed Data which is a 32-element dataset provided by the Case Surveillance Task Force and Surveillance Review and Response Group [17]. The CDC COVID-19 Case Surveillance Restricted Access database was created on April 4, 2020, is updated monthly and the version used in this analysis was last updated on December 4, 2020. It includes de-identified individual level data for 8,405,079 individuals collected across all U.S. states and territories from 1^st^ January to 19^th^ November 2020. The information on each individual in the database was collected using a standard questionnaire (the CDC case report form) [18]. The case definitions for COVID-19 used in this paper are based on the current Council of State and Territorial Epidemiologists case definitions for COVID-19 [19].

#### Additional sources

The National Center for Health Statistics (NCHS) Urban-Rural Classification Scheme (URCS): a classification system for U.S. counties based on population size. It was developed for use in studying and monitoring health disparities across the urban-rural continuum [20]. The most recent (2013) NCHS scheme which we used in this study is based on the 2010 census and the February 2013 office of Management and Budget delineation of metropolitan and micropolitan statistical areas [20].

The CDC Social Vulnerability Index (SVI): created by the Agency for Toxic Substances and Disease Registry Geospatial Research, Analysis & Services Program to “help public health officials and emergency response planners identify and map the communities that will most likely need support before, during, and after a hazardous event” [21]. The CDC SVI categorizes the relative vulnerability of every U.S. census tract or county in four summary themes (Socioeconomic, Minority Status and Language, Household Composition and Disability, Housing Type and Transportation) which rank counties based on their overall vulnerability with higher percentile ranks indicating higher vulnerability [21].

### Patient population

Our inclusion criteria were based on: (1) positive result for SARS-COV-2 infection by a molecular amplification detection test (2) hospitalization for COVID-19 in any of the 4 major census regions (Northeast, West, Midwest and South) designated by the United States Census Bureau [22].

Patients of all ages were included in the analyses.

#### Outcomes and variables

The primary outcome of this study was all cause in-hospital mortality among patients diagnosed with COVID-19, defined by the categorical variable ‘death’ (yes/no) from the COVID-19 Case Surveillance database.

The primary predictors of interest are ‘race’ and ‘neighborhood type’.

The CDC case report form provides multiple options for reporting race/ethnicity. For our analyses, we categorized the variable into 4 different groups as done in previous studies investigating the relationship between race and COVID-19 mortality: Non-Hispanic White, Non-Hispanic Black, Hispanic and Other races (which includes Asian, American Indian/Alaska Native, Native Hawaiian/Other Pacific Islander and multiple ethnicities) [8, 23]. For simplicity, in this paper, we will refer to these racial groups as White, Black, Hispanic and Others respectively.

The Minority Status and Language summary theme of the CDC SVI provides a composite rank for counties based on the proportion of their residents who are non-white and have limited English proficiency (LEP). To generate a variable for ‘neighborhood type’, we grouped the percentiles into quartiles (Q1- low vulnerability, Q2- moderate vulnerability, Q3- high vulnerability, Q4- very high vulnerability) with higher quartiles indicating neighborhoods with a higher proportion of non-white residents and individuals with limited English proficiency.

The socioeconomic summary theme of the CDC SVI provides a composite rank for counties based on the income of residents, proportion who live below poverty, are unemployed or have no high school diploma. To adjust for socioeconomic status, we grouped the percentiles of the socioeconomic summary themes into quartiles (Q1- higher SES, Q2- upper middle SES, Q3- lower middle SES, Q4- lower SES) for our analyses with higher quartiles indicating lower socioeconomic status.

The 2013 NCHS URCS classifies counties into 6 main categories by population size: large central metropolitan, large fringe metropolitan, medium metropolitan, small metropolitan, micropolitan and rural/noncore [20]. For our analyses, county size was defined as metropolitan, micropolitan or rural/noncore [24].

Other variables from the CDC COVID-19 Case Surveillance database which were identified a priori from previous studies as independently associated with mortality from COVID-19 were also included in the analysis. These include sex (male or female) [25], age group (<40, 40–59, 60–79 and 80+) [25, 26], disease severity (non-critical or critical) [27], and presence of comorbidities [5, 28, 29]. We classified disease severity (using the COVID-19 WHO severity classification system) as follows: (1) Critical: Patients with acute respiratory distress syndrome (ARDS), those in the ICU, and those who were mechanically ventilated. (2) Non-critical: Symptomatic patients who do not meet the criteria for critical illness [27]. A patient was considered to have a comorbidity if they had any of the following conditions: Diabetes Mellitus, Hypertension, Severe Obesity (BMI> 40mg/kg^2^) Cardiovascular disease, Chronic Liver disease, Chronic Kidney Disease, Immunosuppressive or autoimmune diseases [18]. A current or previous history of smoking was also classified as a comorbidity [18].

### Statistical analysis

Descriptive statistics for the categorical variables have been presented as frequencies and percentages and were compared using chi-squared tests to evaluate the associations between the categorical variables.

Due to the significant confounding between race and neighborhood type, both variables were not included in the same model (see S4 Table). Multivariable logistic regression models were used to evaluate for disparities in COVID-19 mortality by race and neighborhood type among hospitalized COVID-19 patients on the national level. The models included the primary predictors (model one- race, model two- neighborhood type) and other sociodemographic and health-related variables including age group, sex, socioeconomic status, presence of comorbidity, disease severity, and county size. For model 1, we included an interaction term between race and age group (see S1 Table). We also conducted a subgroup analysis evaluating for racial disparities among patients from neighborhoods with very high vulnerability. In addition, we conducted stratified analyses of racial disparities by (i) disease severity and (ii) time-period (January to June vs July to November).

When interaction terms between (1) race and census region and (2) neighborhood type and census region, were added to the respective multivariable logistic regression models, the race-by-region and neighborhood-by-region interactions were statistically significant (see S2 and S3 Tables respectively).

Therefore, to evaluate, for differences in the pattern of racial and neighborhood disparities across the 4 major census regions, we used different multivariable logistic regression models (one for each census region) which included the outcome of interest (death), the primary predictors (race/ neighborhood type) and other sociodemographic and health-related variables including age group, sex, socioeconomic status, presence of comorbidity, disease severity, and county size. Additionally, based on the results from (ii) above, we conducted a subgroup analysis evaluating patterns of racial disparities across the 4 major census regions in the July to November time-period.

All analyses were performed on the complete cases of the patients by removing all patients with missing information. Missingness for race, neighborhood status and death in the dataset were 24.63%, 1.23% and 29.44% respectively. Detailed information on missing data is provided in the S6 Table. The statistical software R version 4.0.3 (R Foundation for Statistical Computing, Vienna, Austria (2020)) was used to merge the variables in CDC SVI and the NCHS URCS databases to the CDC COVID-19 Case Surveillance database by matching with the county federal information processing codes. All other statistical analyses were conducted using Stata 16.1 (Stata Corp, College Station, TX USA). All tests were two-sided and p values < 0.05 were considered statistically significant.

## Results

**Fig 1:**
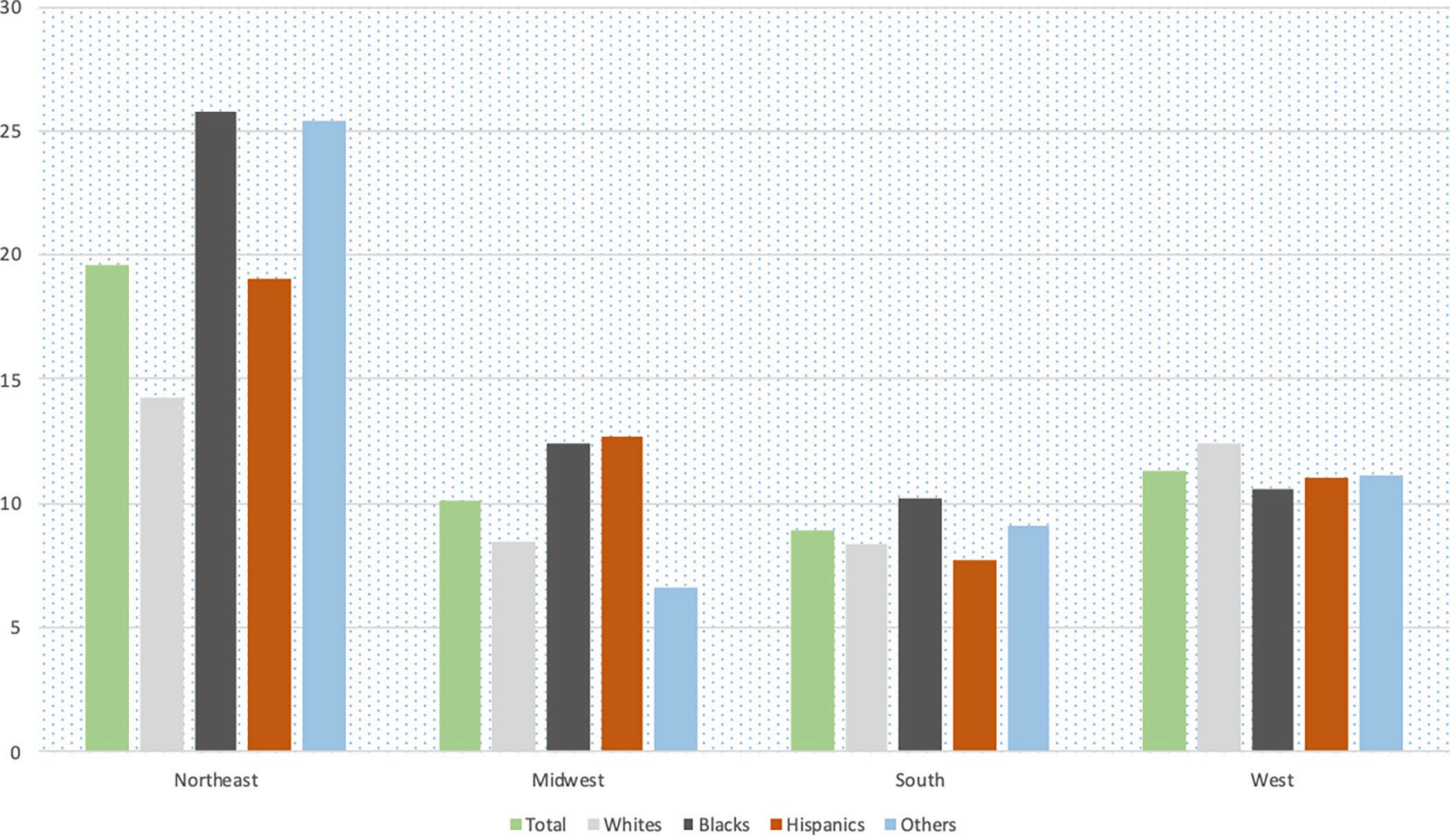
Unadjusted mortality rates (%) per 100 hospitalized COVID-19 patients by racial group among patients <60 years.

**Fig 2:**
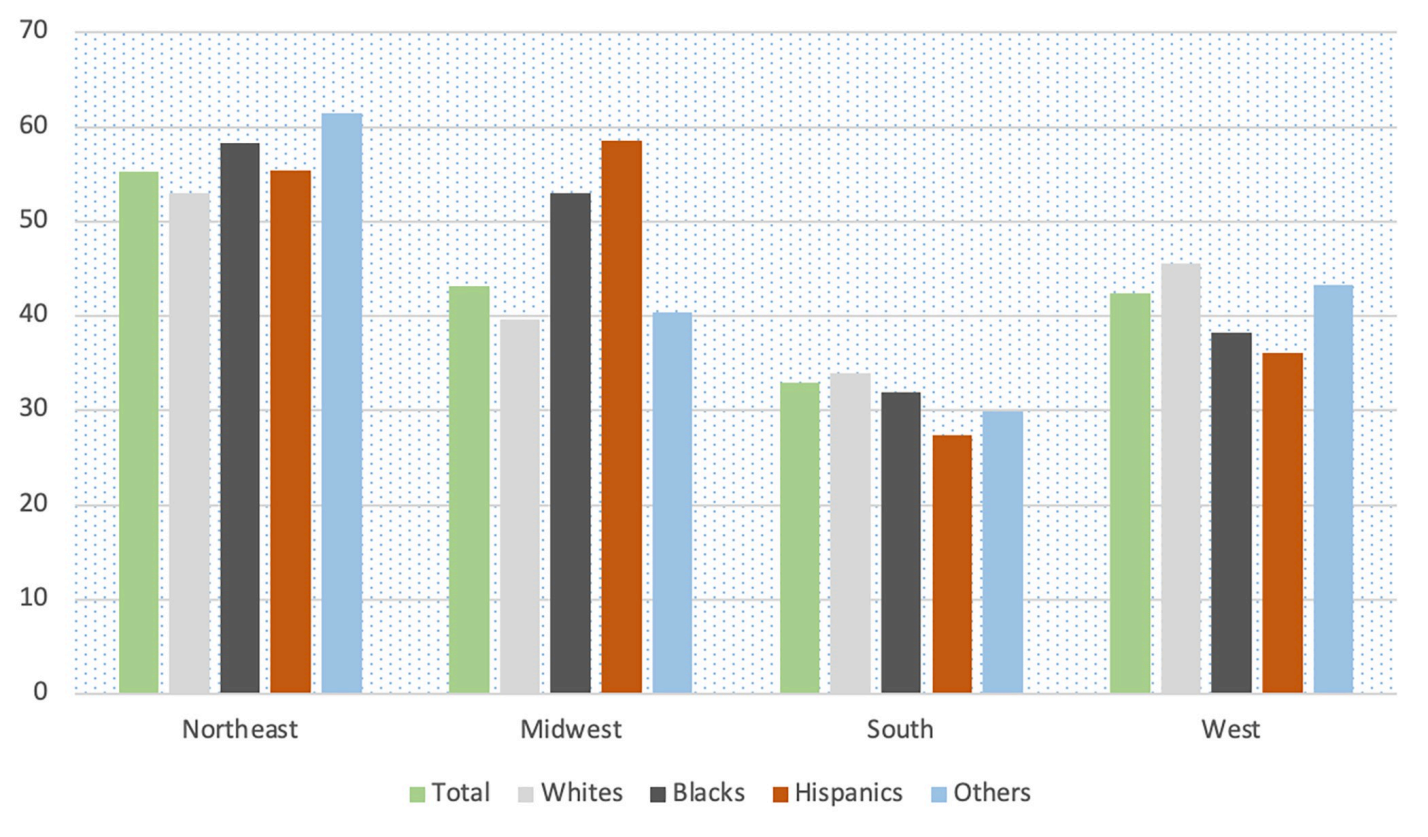
Unadjusted mortality rates (%) per 100 hospitalized COVID-19 patients by racial group among patients 60+ years.

**Fig 3:**
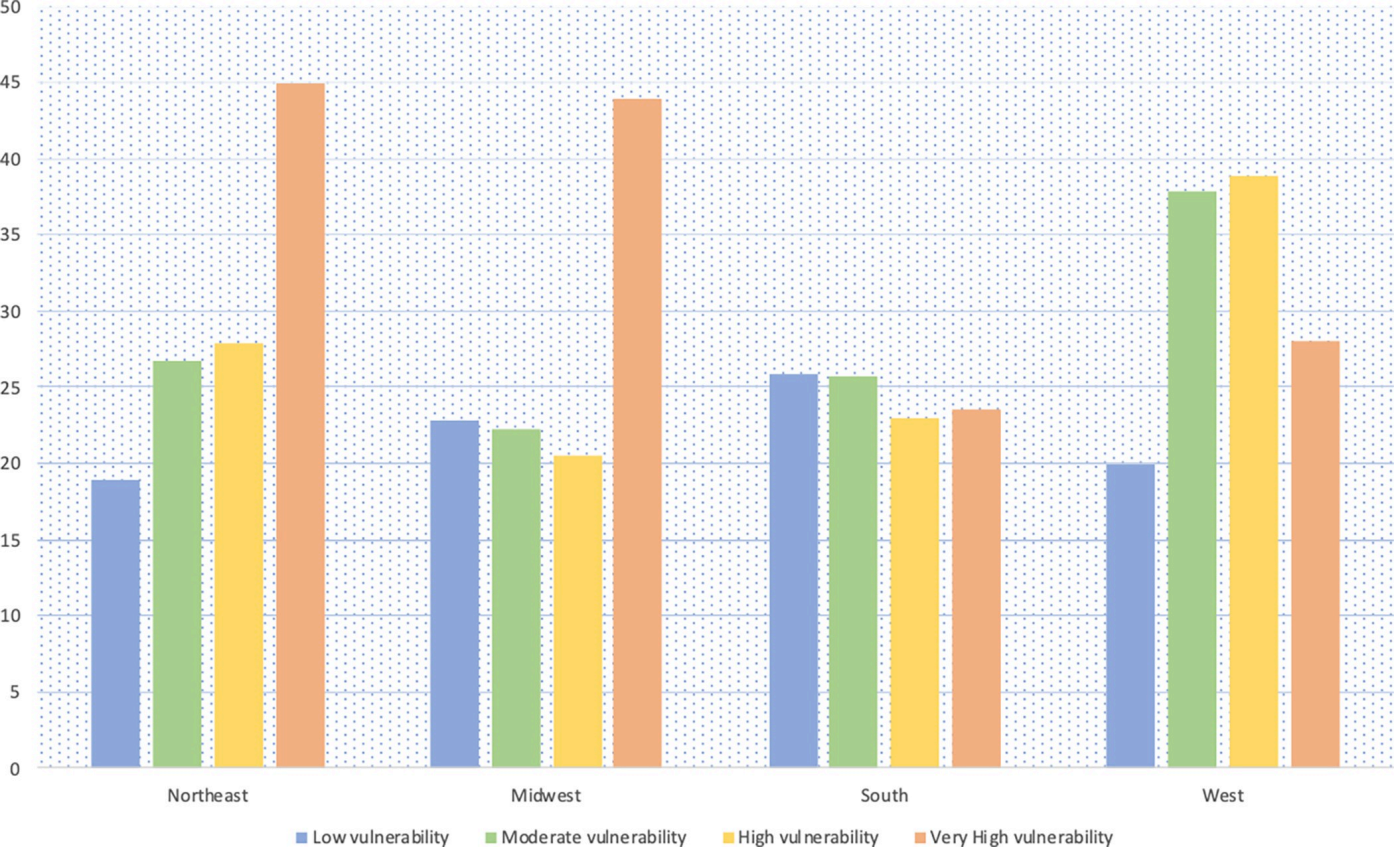
Unadjusted mortality rates (%) per 100 hospitalized COVID-19 patients by neighborhood type.

**Fig 4:**
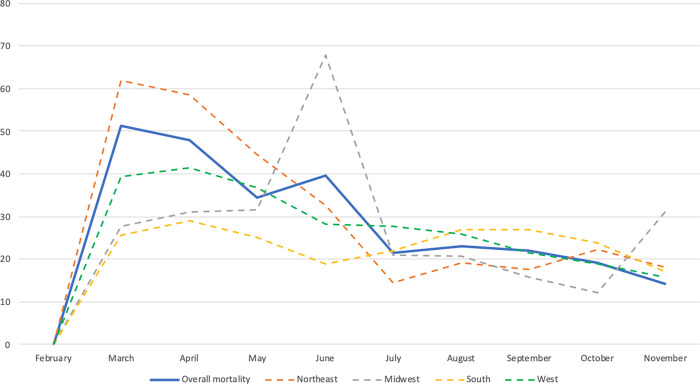
Monthly mortality rates among hospitalized COVID-19 patients by census region.

A total of 106,962 hospitalized COVID-19 patients were included in this analysis. Of these, 55,468 (51.9%) were Whites, 22,589 (21.1%) were Blacks, 20,846 (19.5%) were Hispanics and 8,059 (7.5%) were of other races. Figs 1 and 2 show the mortality rate by racial group in each census region stratified by age (<60 years vs ≥60 years), all of which were highest in the Northeast. In the Northeast, mortality rates among Blacks <60 years was about twice the mortality rates in Whites; among those ≥ 60 years, the mortality rates were about even. Fig 3 shows the mortality rate by neighborhood type. In the Northeast and Midwest, patients from neighborhoods with very high vulnerability had the highest mortality rates. National mortality rates during the study were highest in April and the mortality rates trended downwards towards November (Fig 4).

Over three-fifths of the hospitalized cases were ≥60 years old and about one-fifth were over 80 years. The sex distribution of the hospitalized population was about even. Most patients were from metropolitan counties (93,673, 87.6%), had at least one comorbidity (92,809, 86.8%), and about one-third (33,845, 31.6%) were in critical condition. The mortality rate among all hospitalized COVID-19 patients during the study period was about 33% (35,569/106,962). Detailed characteristics of the study population by racial category is shown in Table 1.

**Table 1:** Demographic and clinical characteristics of hospitalized COVID-19 patients by racial category (N = 106,962) for the complete case analysis.

Demographic and Clinical variables	Total	White	Black	Hispanic	Others	p-value
	106,962 (%)	55,468 (%)	22,589 (%)	20,846 (%)	8,059 (%)	
**Sex**						
Female	50,510 (47.22)	25,967 (46.81)	11,716 (51.87)	9,068 (43.50)	3,759 (46.64)	<0.0001
Male	56,452 (52.78)	29,501 (53.19)	10,873 (48.13)	11,778 (56.50)	4,300 (53.36)	
**Age Group**						
<40 years	13,278 (12.41)	4,094 (7.38)	3,048 (13.49)	4,887 (23.44)	1,249 (15.50)	<0.0001
40–59 years	28,164 (26.33)	10,625 (19.16)	6,877 (30.44)	8,272 (39.68)	2,390 (29.66)	
60–79 years	44,154 (41.28)	25,157 (45.35)	9,638 (42.67)	6,088 (29.20)	3,271 (40.59)	
80+ years	21,366 (19.98)	15,592 (28.11)	3,026 (13.40)	1,599 (7.67)	1,149 (14.26)	
**Comorbidities**						
Absent	14,153 (13.23)	6,717 (12.11)	2,192 (9.70)	3,938 (18.89)	1,306 (16.21)	<0.0001
Present	92,809 (86.77)	48,751 (87.89)	20,397 (90.30)	16,908 (81.11)	6,753 (83.79)	
**Disease Severity**						
Non-critical	73,117 (68.36)	39,290 (70.83)	15,138 (67.01)	13,624 (65.36)	5,065 (62.85)	<0.0001
Critical	33,845 (31.64)	16,178 (29.17)	7,451 (32.99)	7,222 (34.64)	2,994 (37.15)	
**Socioeconomic Status (SES)**						
Quartile 1 (Higher SES)	33,993 (31.78)	21,461 (38.69)	4,455 (19.72)	5,385 (25.83)	2,692 (33.40)	<0.0001
Quartile 2 (Upper middle SES)	29,986 (28.03)	15,509 (27.96)	5,951 (26.34)	6,320 (30.32)	2,206 (27.37)	
Quartile 3 (Lower middle SES)	32,648 (30.52)	14,265 (25.72)	8,697 (38.50)	7,083 (33.98)	2,603 (32.30)	
Quartile 4 (Lower SES)	10,335 (9.66)	4,233 (7.63)	3,486 (15.43)	2,058 (9.87)	558 (6.92)	
**Neighborhood Type**						
Quartile 1 (low vulnerability)	5,351 (5.00)	5,210 (9.39)	40 (0.18)	23 (0.11)	78 (0.97)	<0.0001
Quartile 2 (moderate vulnerability)	8,986(8.40)	7,641 (13.78)	812 (3.59)	331 (1.59)	202 (2.51)	
Quartile 3 (high vulnerability)	24,971 (23.35)	16,550 (29.84)	4,696 (20.79)	2,681 (12.86)	1,044 (12.95)	
Quartile 4 (very high vulnerability)	67,654 (63.25)	26,067 (46.99)	17,041 (75.44)	17,811 (85.44)	6,735 (83.57)	
**Census Region**						
Northeast	40,326 (37.70)	17,672 (31.86)	7,856 (34.78)	11,054 (53.03)	3,744 (46.46)	<0.0001
Midwest	36,889 (34.49)	23,120 (41.68)	7,413 (32.82)	4,019 (19.28)	2,337 (29.00)	
South	20,111 (18.80)	10,663 (19.22)	6,400 (28.33)	2,436 (11.69)	612 (7.59)	
West	9,636 (9.01)	4,013 (7.23)	920 (4.07)	3,337 (16.01)	1,366 (16.95)	
**County Size**						
Metropolitan	93,673 (87.58)	45,454 (81.95)	20,901 (92.53)	19,558 (93.82)	7,760 (96.29)	<0.0001
Micropolitan	8,419 (7.87)	6,163 (11.11)	1,103 (4.88)	916 (4.39)	237 (2.94)	
Rural/Noncore	4,870 (4.55)	3,851 (6.94)	585 (2.59)	372 (1.78)	62 (0.77)	
**Death**						
No	71,393 (66.75)	36,295 (65.43)	14,851 (65.74)	14,998 (71.95)	5,249 (65.13)	<0.0001
Yes	35,569 (33.25)	19,173 (34.57)	7,738 (34.26)	5,848 (28.05)	2,810 (34.87)	

In the unadjusted model, Hispanics had lower odds of dying [OR 0.74, 95% CI (0.71, 0.76)] while there was no difference in the mortality odds between Blacks and White patients [OR 0.99, 95% CI (0.95, 1.02)]. However, after adjusting for age group, sex, presence of comorbidity, disease severity, county size, and socioeconomic status, Blacks and Hispanics had higher odds of dying [OR of 1.20, 95% CI (1.15, 1.25) and 1.23, 95% CI (1.17, 1.28) respectively] compared with White patients. In the subgroup analysis among residents from neighborhoods with very high vulnerability, Blacks had the highest mortality odds of all the racial groups. In both unadjusted and adjusted analyses, the mortality odds among patients from neighborhoods with moderate and high vulnerability did not differ significantly from mortality odds of those from neighborhoods with low vulnerability. Patients from neighborhoods with very high vulnerability however had the highest mortality odds [Adjusted OR 2.08, 95% CI (1.91, 2.26)]. These results are shown in Table 2A and 2B. The adjusted mortality odds for the other covariates are almost identical between Model 1 (race as primary predictor) and Model 2 (neighborhood type as primary predictor). Results of the fully adjusted model are shown in Table 3. The model assessing for effect modification of the relationship between race and mortality by age group showed that among patients <40 years, Blacks and Hispanics had higher mortality odds than Whites and this trend is consistent in the 40–79-year age group. However, in the 80+ year age group, Whites had the highest mortality odds (see S1 Table).

**Table 2:** a: Logistic regression results for racial disparities in mortality among hospitalized COVID-19 patients. b: Logistic regression results for racial disparities in mortality among hospitalized COVID-19 patients from neighborhoods with very high vulnerability.

**(a)**		
**Model 1 (reference is White)**	**Unadjusted OR**	^*^ **Adjusted OR,**
	**(95% CI)**	**(95% CI** ^*^ **)**
Black	0.99 (0.95, 1.02)	1.20 (1.15, 1.25)
Hispanic/Latino	0.74 (0.71, 0.76)	1.23 (1.17, 1.28)
Other races	1.01 (0.96, 1.06)	1.22 (1.15, 1.30)
**Model 2 (reference is low vulnerability)**		
Quartile 2 (moderate vulnerability)	1.02 (0.94, 1.10)	0.98 (0.89, 1.08)
Quartile 3 (high vulnerability)	1.03 (0.96, 1.11)	0.98 (0.90, 1.07)
Quartile 4 (very high vulnerability)	2.11 (1.97, 2.25)	2.08 (1.91, 2.26)
**(b)**		
**Reference is White**	**Unadjusted OR**	^*^ **Adjusted OR,**
	**(95% CI)**	**(95% CI** ^*^ **)**
Black	0.80 (0.77, 0.83)	1.06 (1.01, 1.11)
Hispanic/Latino	0.56 (0.54, 0.58)	0.96 (0.91, 1.01)
Other races	0.79 (0.75, 0.83)	0.98 (0.92, 1.05)

Model 1: Racial category is primary predictor. Model 2: Neighborhood type is primary predictor.

*Adjusted for age group, sex, presence of comorbidity, disease severity, socioeconomic status, and county size.

**Table 3:** Results of the fully adjusted multivariable logistic regression model.

Variables	Model 1 Adjusted OR, 95% CI*	Model 2 Adjusted OR, 95% CI*
**Racial Group (Reference category is White)**		
Black	1.20 (1.15, 1.25)	-
Hispanic/Latino	1.23 (1.17, 1.28)	-
Other races	1.22 (1.15, 1.30)	-
**Neighborhood type (reference is low vulnerability)**		
Quartile 2 (moderate vulnerability)	-	0.98 (0.89, 1.08)
Quartile 3 (high vulnerability)	-	0.98 (0.90, 1.07)
Quartile 4 (very high vulnerability)	-	2.08 (1.91, 2.26)
**Sex (Reference category is Female)**		
Male	1.38 (1.34, 1.43)	1.38 (1.34, 1.43)
**Age Group (Reference category is <40 years)**		
40–59 years	2.94 (2.69, 3.21)	2.95 (2.70, 3.22)
60–79 years	9.01 (8.28, 9.80)	9.11 (8.38, 9.91)
80+ years	36.16 (33.09, 39.51)	35.93 (32.92, 39.22)
**Presence of comorbidities (Reference category is Absent)**		
Present	3.03 (2.84, 3.23)	2.93 (2.75, 3.12)
**Disease severity (Reference category is non-critical)**		
Critical	5.82 (5.64, 6.02)	5.92 (5.73, 6.12)
**County size (Reference category is Metropolitan)**		
Micropolitan	0.42 (0.39, 0.45)	0.58 (0.54, 0.62)
Rural/Noncore	0.39 (0.35, 0.41)	0.47 (0.35, 0.56)
**Socioeconomic status (Reference category is Higher SES)**		
Quartile 2 (Upper middle SES)	1.02 (0.98, 1.07)	0.97 (0.93, 1.01)
Quartile 3 (lower middle SES)	1.45 (1.40, 1.51)	1.30 (1.25, 1.35)
Quartile 4 (lower SES)	1.69 (1.59, 1.80)	1.46 (1.37, 1.55)

*Adjusted for age group, sex, presence of comorbidity, disease severity, socioeconomic status, and county size.

In the analysis stratified by disease severity, among patients in critical condition, Black and Hispanic patients had higher odds of dying [OR of 1.40, 95% CI (1.32, 1.50) and 1.56, 95% CI (1.46, 1.67) respectively] compared with White patients (Table 4A). In the analysis stratified by time-period, there was no difference in mortality between Black, Hispanic, and White patients between January and June. Between July and November, compared to White patients, Black patients had higher odds of mortality [OR of 1.10, 95% CI (1.02, 1.19)] and these findings were driven by disparities in the Midwest. There was however no difference in the overall mortality odds between Hispanic and White patients [OR of 1.04, 95% CI (0.94, 1.14)] during this period. These results are shown in Table 4B and 4C.

**Table 4:** a: Logistic regression results for racial disparities in mortality among hospitalized COVID-19 patients stratified by disease severity. b: Logistic regression results for racial disparities in mortality among hospitalized COVID-19 patients, stratified by periods of the pandemic. c: Comparing disparities in mortality among hospitalized COVID-19 patients in July to November, across the 4 census regions.

**(a)**				
**Reference is White**	**Non-Critical Unadjusted OR**	**Non-Critical** ^*^Adjusted OR,	**Critical Unadjusted OR**	**Critical** *Adjusted OR,
	**(95% CI)**	**(95% CI** * **)**	**(95% CI)**	**(95% CI** * **)**
Black	0.73 (0.69, 0.76)	1.09 (1.03, 1.15)	1.35 (1.28, 1.43)	1.40 (1.32, 1.50)
Hispanic/Latino	0.41 (0.39, 0.43)	0.97 (0.91, 1.04)	1.10 (1.04, 1.16)	1.56 (1.46, 1.67)
Other races	0.71 (0.66, 0.76)	1.09 (1.01, 1.19)	1.22 (1.13, 1.32)	1.44 (1.31, 1.57)
**(b)**				
**Reference is White**	**January to June Unadjusted OR**	**January to June** *Adjusted OR,	**July to November Unadjusted OR**	**July to November** *Adjusted OR,
	**(95% CI)**	**(95% CI** * **)**	**(95% CI)**	**(95% CI** * **)**
Black	0.86 (0.82, 0.89)	0.98 (0.93, 1.03)	0.80 (0.75, 0.85)	1.10 (1.02, 1.19)
Hispanic/Latino	0.58 (0.56, 0.61)	0.97 (0.92, 1.03)	0.57 (0.53, 0.62)	1.04 (0.94, 1.14)
Other races	0.92 (0.87, 0.98)	1.10 (1.02, 1.18)	0.68 (0.61, 0.75)	0.92 (0.81, 1.04)
**(c)**				
**Racial Group (White is reference category)**	**Northeast OR** *	**Midwest OR** *	South OR*,	**West, OR** *
	**(95% CI)**	**(95% CI)**	**(95% CI)**	**(95% CI)**
Black	1.26 (0.93, 1.70)	1.19 (1.05, 1.35)	1.05 (0.95, 1.18)	0.98 (0.74, 1.30)
Hispanic/Latino	0.74 (0.55, 0.99)	1.22 (1.02, 1.45)	0.95 (0.77, 1.17)	0.95 (0.79, 1.13)
Other races	1.28 (0.93, 1.76)	0.74 (0.59, 0.91)	0.97 (0.70, 1.34)	0.90 (0.72, 1.13)

(a) *Adjusted for age group, sex, presence of comorbidity, disease severity, socioeconomic status, and county.

(b and c) *Adjusted for age group, sex, presence of comorbidity, disease severity, socioeconomic status, and county size.

In the Midwest, Blacks [OR 1.27, 95% CI (1.18, 1.37)] and Hispanics [OR 1.99, 95% CI (1.80, 2.20)] had higher odds of mortality compared with Whites. In the Northeast, South and West, Hispanics had lower mortality odds compared to Whites while there was no difference in mortality odds between Blacks and Whites. In the Northeast and Midwest, patients from neighborhoods with very high vulnerability had the highest mortality odds. In the South, patients from neighborhoods in quartile 3 (high vulnerability) had lower mortality odds than those in quartile 1 (low vulnerability). In the West, most patients, 8995/9636 (93.4%) are from neighborhoods with very high vulnerability (quartile 4) and as a result, there are very wide confidence intervals around the estimates. These results are summarized in Table 5. The neighborhood distribution of patients in the West is shown in the S5 Table.

**Table 5:** Comparing disparities in mortality among hospitalized COVID-19 patients across the 4 census regions.

Racial Group (White is reference category)	Northeast OR	Midwest OR	South OR,	West, OR
	(95% CI)	(95% CI)	(95% CI)	(95% CI)
Black	1.06 (0.99, 1.14)	1.27 (1.18, 1.37)	1.07 (0.97, 1.16)	0.97 (0.79, 1.18)
Hispanic/Latino	0.80 (0.74, 0.85)	1.99 (1.80, 2.20)	0.76 (0.66, 0.89)	0.82 (0.72, 0.94)
Other races	1.15 (1.05, 1.26)	0.98 (0.87, 1.11)	0.94 (0.75, 1.20)	0.83 (0.70, 0.97)
**Neighborhood type (reference is low vulnerability)**				
Quartile 2 (moderate vulnerability)	2.37 (1.66, 3.38)	0.89 (0.79, 0.99)	0.96 (0.78, 1.17)	2.30 (0.17, 31.82)
Quartile 3 (high vulnerability)	2.81 (2.03, 3.89)	0.85 (0.76, 0.95)	0.82 (0.69, 0.98)	1.25 (0.10, 15.45)
Quartile 4 (very high vulnerability)	4.79 (3.48, 6.61)	2.85 (2.55, 3.19)	0.96 (0.81, 1.14)	0.96 (0.08, 11.88)

*Adjusted for age group, sex, presence of comorbidity, disease severity, socioeconomic status, and county size.

## Discussion

The association between race and neighborhood with COVID-19 mortality is complex and nuanced. Although there are differences overall, they are driven by disparities in time-period and specific geographical regions. When adjusted for age group, sex, presence of comorbidity, disease severity, socioeconomic status, and county size, hospitalized Blacks and Hispanics were more likely to die compared with Whites, but these differences are driven by disparities in the Midwest and the July to November time-period. We also found that patients from high vulnerability neighborhoods (those with the highest proportion of racial minorities and individuals with limited English proficiency) had the highest odds of mortality among hospitalized COVID-19 patients, though these differences were limited to the Northeast and Midwest.

Overall, disparities in COVID-19 in-hospital mortality by race were driven by differences in the Midwest–there were no differences in the West, South, Northeast. Additionally, between January and June, there were no disparities in COVID-19 mortality between Whites, Blacks and Hispanics. This is consistent with findings from retrospective cohort studies conducted in Louisiana, Georgia (both Southern states) and California (western state) during the January to June time-period which found no difference in mortality rates between Blacks and Whites among hospitalized COVID-19 patients [11, 30, 31]. It contrasts, however, with the findings of the study conducted in New York by Ogedegbe et al. which found that Blacks were less likely than Whites to die from COVID-19 among hospitalized patients [12]. Their study was conducted using information from patients in a single health system which mainly included patients from Manhattan, Brooklyn, Queens, and Long Island and may not completely represent the characteristics of the entire New York population [12].

Several studies and reports in the Mid-West during periods of ICU bed shortage documented a higher overall mortality from COVID-19 among Blacks and Hispanics compared to Whites [32–34]. The finding of a higher overall mortality rate in Blacks [9, 32] but comparable in-hospital mortality rates between Blacks and Whites across multiple studies [11–13], suggest that factors related to care access contribute to the racial disparities seen in COVID-19 mortality. Reduced access to care is a multifaceted problem and could be due to underinsurance, geographic disparities in hospital location leading long transportation and emergency room wait times, all of which affect healthcare seeking behavior [35]. In addition, distrust of healthcare professionals and perceived racial discrimination within the healthcare system has been shown to significantly affect the healthcare seeking behavior of racial minorities [36].

Our study goes further to show that racial disparities in mortality exist even among hospitalized COVID-19 patients in the Midwest. This suggests that other in-hospital factors may contribute to the observed racial disparities in mortality. Previous studies conducted in other emergency settings have identified factors including physician race/ethnic case mix and implicit bias among healthcare workers as responsible for racial disparities in health outcomes [37, 38]. In multiple emergency settings physician treatment recommendations have been found to vary by race. A study conducted among patients with acute coronary syndrome found that Black patients were less likely than Whites to be referred for cardiac catheterization [39]. These disparities are worsened during periods of hospital overcrowding and bed shortage (as occurred in the Midwest during the early months of the pandemic), resulting in racial minorities receiving poorer quality care [40]. The importance of race as a predictor of in-hospital COVID-19 mortality is made more compelling by the finding that Blacks had a higher likelihood of death compared to Whites even within the very high vulnerability neighborhoods, and after stratification by disease severity. Although Hispanics in non-critical condition were just as likely as to die as Whites, Hispanics in critical condition were much more likely to die compared to Whites. As patients in critical condition are more likely to require a higher level of care, these findings further highlight the racial inequities in care that occur within the hospital.

Race is a complex concept in public health, and its categories may be too broad for appropriate nuance. Although there have been arguments that higher comorbidity levels are responsible for the higher level of mortality seen among Hispanics and Blacks, Qeadan et al. found in their study, which was stratified by comorbidity index, that mortality in Blacks was consistently higher than that of Whites. Hispanics however had a lower risk of mortality [41]. This is demonstrated again in our study, where compared to Whites, Hispanics had significantly lower mortality odds in the Northeast, South and West. As early as the 1980’s, researchers found that the health status of Hispanics in the southwestern states of the U.S. was closer to the health status of Whites than that of Blacks [42]. Studies on the ‘Hispanic paradox’ have even found better health outcomes among Hispanics compared to their White counterparts in the U.S. [43–45]. The yet unmeasured factors accounting for these better outcomes may explain why Hispanics in the South and West have lower odds of mortality than the Blacks in our study. The Hispanic population is also not monolithic and health outcomes vary between different Hispanic subpopulations [46]. The different compositions Hispanic populations across the U.S. may in part explain the difference in COVID-19 mortality observed in our study between Hispanics in the Midwest and those in the other census regions.

Disparities in COVID-19 in-hospital mortality by neighborhood vulnerability were driven by differences in the Midwest and Northeast–there were no differences in the West and South. These disparities may be due to inequities in healthcare resource allocation between hospitals in different neighborhoods; inequities which were worsened by the hospital overcrowding and ICU bed shortage that occurred disproportionately in the Northeast and Midwest during the peak of the pandemic [3, 6, 10]. The optimum hospital bed occupancy rate is estimated to be between 80%-85% with discernible mortality risks above these rates [47, 48]. Data from the American Hospital Association showed hospital bed occupancy rates of over 90% in the Northeast and Midwest during our study period, but not for the South or West [49]. In New York City, Manhattan which has the highest proportion of White residents and the most equipped medical centers recorded the lowest number of COVID-19 deaths [10, 50]. These hospitals were properly staffed and had access to experimental drugs like Remdesivir and life-saving devices like heart-lung bypass machines [50]. Conversely, the Bronx which has the highest proportion of minority residents and less equipped hospitals recorded the highest number of COVID-19 deaths per 100,000 population [10]. The understaffing and inadequate access to high-technology diagnostic and therapeutic procedures in these hospitals were exacerbated by the increased demands of the pandemic resulting in disproportionately higher mortality [51]. A recent report suggests that increased access to novel therapeutics for patients with limited English proficiency can help close the language-based disparity gaps in patient outcomes within the acute care setting [52].

We found an overall mortality rate of 33% among hospitalized COVID-19 patients. The mortality rate in our study is higher than the 20.3% mortality rate reported in the multi-state retrospective study conducted by Yehia et al. across 92 hospitals in the U.S. [13]. Their study however collected data from only 2 hospitals in New York where mortality from COVID-19 was the highest during the February to May study period and thus likely underestimated the overall mortality rate. The authors also reported no difference in mortality by race which contrasts with our study’s findings. The 92 hospitals in their study were however located in only 12 states and over a third of them were from the South alone (where we found no difference in mortality between Blacks and Whites).

Our study has limitations. (1) Like all studies conducted using secondary data, our ability to adjust for confounding depends on the number of variables in our dataset and the results of our analysis are subject to the accuracy of the information provided by the different reporting authorities. For example, we were unable to adjust for hospital and provider characteristics in our analyses. As such, residual confounding cannot be eliminated. (2) Socioeconomic status was measured at zip-code level and may not reflect individual factors. (3) The CDC COVID-19 Case Surveillance database contained varying amounts of missing data, some of which could not be assumed to be missing completely at random. Missing at random is an assumption (MAR) and missing not at random (MNAR) cannot be ruled out empirically [53]. Analysis of data that are MNAR do not however guarantee that the study estimates will be biased; it only implies that we cannot correct for bias if present [53]. Studies have also shown that even when the exposure and/or confounders are MNAR, complete case analysis is a valid approach [54]. We however used the multiple imputation with chained equations (MICE) algorithm on our data and conducted analyses on the imputed datasets to test the robustness of the results from our complete case analyses (see S7 Table). For our primary objective, the results from the imputation model are similar (albeit attenuated) to those from the complete case analyses.

The biggest strength of our study is its large sample size which reduces the variability in our effect estimates. The data used in this study was collected from all states and territories in the United States. Hence, despite its limitations, our study provides important epidemiological data on Blacks and Hispanics in the context of the COVID-19 pandemic.

## Conclusion

Our study findings show that among hospitalized COVID-19 patients in the United States, Blacks and Hispanics have an overall higher odd of mortality in the Midwest, and residents of neighborhoods with the highest proportion of racial minorities and individuals with limited English proficiency have higher odds of mortality in the Northeast and Midwest. These results suggest that efforts to curb healthcare disparities, eliminate structural racism and reduce inequity in resource allocation in the healthcare setting have largely been unsuccessful. The timing of these findings in the middle of a global pandemic presents a unique opportunity to address these issues.

## Supporting information

S1 Table Regression model with interaction terms between racial category and age group. (DOCX)

S2 Table Regression model with interaction terms between racial category and census region. (DOCX)

S3 Table Regression model with interaction terms between neighborhood type and census region. (DOCX)

S4 Table Regression model including both primary predictors- race and neighborhood type. (DOCX)

S5 Table Distribution of neighborhood type in the west. (DOCX)

S6 Table Missing data table. (DOCX)

S7 Table Multiple imputation models. (DOCX)

S1 Data Link to datasets used in the analyses. (DOCX)
