# Supplementary material for: Racial and neighborhood disparities in mortality among hospitalized COVID-19 patients in the United States: An analysis of the CDC case surveillance database
Source: PLOS Glob Public Health. 2022 Nov 16;2(11):e0000701. doi: 10.1371/journal.pgph.0000701 (PMC10022015; doi:10.1371/journal.pgph.0000701)
Supplement: S1 Table — (DOCX) [file pgph.0000701.s001.docx]

**Model with interaction terms between racial category and age group**

-----------------------------------------------------------------------------------------------------

death_yn | Odds ratio Std. err. z P>|z| [95% conf. interval]

------------------------------------+----------------------------------------------------------------

racial_cat |

Black, Non-Hispanic | 1.480496 .1730817 3.36 0.001 1.177322 1.861742

Hispanic/Latino | 1.405508 .1530913 3.13 0.002 1.13532 1.739996

Other races | 1.100509 .1814239 0.58 0.561 .7966531 1.520261

|

age_cat |

40 - 59 Years | 2.936672 .2739225 11.55 0.000 2.446011 3.525759

60 - 79 Years | 10.0118 .8951125 25.77 0.000 8.402529 11.92929

80+ Years | 41.7149 3.757033 41.42 0.000 34.96456 49.76847

|

racial_cat#age_cat |

Black, Non-Hispanic#40 - 59 Years | .9653263 .1207356 -0.28 0.778 .7554616 1.233491

Black, Non-Hispanic#60 - 79 Years | .8013485 .0961475 -1.85 0.065 .633421 1.013796

Black, Non-Hispanic#80+ Years | .689577 .0862925 -2.97 0.003 .5395911 .8812533

Hispanic/Latino#40 - 59 Years | 1.003075 .1173337 0.03 0.979 .7975629 1.261542

Hispanic/Latino#60 - 79 Years | .8839415 .100458 -1.09 0.278 .7074347 1.104487

Hispanic/Latino#80+ Years | .6665698 .0831067 -3.25 0.001 .5220588 .851083

Other races#40 - 59 Years | 1.268655 .2239581 1.35 0.178 .8975931 1.793114

Other races#60 - 79 Years | 1.096533 .186612 0.54 0.588 .7855267 1.530672

Other races#80+ Years | 1.130238 .2028887 0.68 0.495 .7950071 1.606826

|

sex | 1.383038 .0218434 20.53 0.000 1.340882 1.42652

medcond_yn | 3.031899 .0981658 34.26 0.000 2.845475 3.230536

|

critical |

Critical | 5.835846 .0976825 105.39 0.000 5.647498 6.030475

|

county_size |

Micropolitan | .4189198 .0135819 -26.84 0.000 .3931279 .4464039

Rural/Noncore | .3768406 .0159761 -23.02 0.000 .3467936 .409491

|

ses_svi |

25th to 50th percentile | 1.02525 .0211509 1.21 0.227 .9846217 1.067554

50th to 75th percentile | 1.455389 .0291119 18.76 0.000 1.399435 1.513581

>75th percentile | 1.690449 .0514524 17.25 0.000 1.592553 1.794363

|

_cons | .0074477 .0006977 -52.30 0.000 .0061984 .0089488
