# Supplementary material for: Racial and neighborhood disparities in mortality among hospitalized COVID-19 patients in the United States: An analysis of the CDC case surveillance database
Source: PLOS Glob Public Health. 2022 Nov 16;2(11):e0000701. doi: 10.1371/journal.pgph.0000701 (PMC10022015; doi:10.1371/journal.pgph.0000701)
Supplement: S2 Table — (DOCX) [file pgph.0000701.s002.docx]

**Model with interaction terms between racial category and census region**

-----------------------------------------------------------------------------------------------

death_yn | Odds ratio Std. err. z P>|z| [95% conf. interval]

------------------------------+----------------------------------------------------------------

racial_cat |

Black, Non-Hispanic | 1.262115 .0428762 6.85 0.000 1.180816 1.349012

Hispanic/Latino | .9808673 .0317844 -0.60 0.551 .9205081 1.045184

Other races | 1.306255 .0582332 5.99 0.000 1.196964 1.425525

|

region |

Midwest | .5449449 .0138435 -23.90 0.000 .5184766 .5727644

South | .3265445 .0110545 -33.06 0.000 .3055813 .3489458

West | .4239676 .0186866 -19.47 0.000 .3888799 .4622213

|

racial_cat#region |

Black, Non-Hispanic#Midwest | 1.009184 .0484649 0.19 0.849 .9185277 1.108787

Black, Non-Hispanic#South | .8152244 .0445028 -3.74 0.000 .7325046 .9072854

Black, Non-Hispanic#West | .4874349 .0504028 -6.95 0.000 .3980145 .5969451

Hispanic/Latino#Midwest | 1.74771 .0991391 9.84 0.000 1.563814 1.953233

Hispanic/Latino#South | .9255048 .0741846 -0.97 0.334 .7909516 1.082948

Hispanic/Latino#West | .7175204 .0514611 -4.63 0.000 .6234268 .8258155

Other races#Midwest | .6974345 .0533679 -4.71 0.000 .6003011 .8102847

Other races#South | .7454631 .09608 -2.28 0.023 .5790524 .9596975

Other races#West | .5978169 .0557611 -5.52 0.000 .4979351 .7177342

|

sex | 1.35991 .0218832 19.10 0.000 1.317689 1.403484

|

age_cat |

40 - 59 Years | 3.009645 .1356224 24.45 0.000 2.75523 3.287552

60 - 79 Years | 9.504396 .4146083 51.62 0.000 8.725549 10.35276

80+ Years | 38.24244 1.753575 79.47 0.000 34.95541 41.83855

|

medcond_yn | 2.59482 .0848247 29.17 0.000 2.433781 2.766515

|

critical |

Critical | 6.384464 .1105758 107.04 0.000 6.171377 6.604909

|

county_size |

Micropolitan | .547306 .0182862 -18.04 0.000 .512614 .5843459

Rural/Noncore | .5183806 .0224946 -15.14 0.000 .4761149 .5643983

|

ses_svi |

25th to 50th percentile | 1.07636 .0226118 3.50 0.000 1.032942 1.121604

50th to 75th percentile | 1.981517 .0424179 31.95 0.000 1.9001 2.066424

>75th percentile | 2.221932 .0725436 24.45 0.000 2.084203 2.368763

|

_cons | .0127 .0007066 -78.48 0.000 .0113879 .0141632
