# Supplementary material for: Racial and neighborhood disparities in mortality among hospitalized COVID-19 patients in the United States: An analysis of the CDC case surveillance database
Source: PLOS Glob Public Health. 2022 Nov 16;2(11):e0000701. doi: 10.1371/journal.pgph.0000701 (PMC10022015; doi:10.1371/journal.pgph.0000701)
Supplement: S3 Table — (DOCX) [file pgph.0000701.s003.docx]

**Model with interaction terms between neighborhood type and census region**

--------------------------------------------------------------------------------------------------

death_yn | Odds ratio Std. err. z P>|z| [95% conf. interval]

---------------------------------+----------------------------------------------------------------

minority_svi |

25th to 50th percentile | 2.102745 .3623124 4.31 0.000 1.500102 2.947491

50th to 75th percentile | 2.575289 .4020161 6.06 0.000 1.896483 3.497059

>75th percentile | 5.059066 .777604 10.55 0.000 3.743142 6.83761

|

region |

Midwest | 1.525315 .2429698 2.65 0.008 1.116273 2.084245

South | 1.334504 .2311218 1.67 0.096 .9503881 1.873868

West | 1.489788 1.800935 0.33 0.742 .1393628 15.92583

|

minority_svi#region |

25th to 50th percentile#Midwest | .4411515 .0801086 -4.51 0.000 .3090426 .629734

25th to 50th percentile#South | .5193299 .1042638 -3.26 0.001 .3503894 .7697253

25th to 50th percentile#West | .9505419 1.222496 -0.04 0.969 .0764254 11.82238

50th to 75th percentile#Midwest | .3376768 .0556273 -6.59 0.000 .2444996 .4663632

50th to 75th percentile#South | .3498647 .0625891 -5.87 0.000 .2463907 .4967936

50th to 75th percentile#West | .7028994 .8527922 -0.29 0.771 .0651889 7.579016

>75th percentile#Midwest | .5452495 .0878467 -3.76 0.000 .3976086 .7477128

>75th percentile#South | .2057609 .0360644 -9.02 0.000 .1459385 .2901054

>75th percentile#West | .1819521 .2200443 -1.41 0.159 .0170041 1.946974

|

sex | 1.365099 .0221787 19.16 0.000 1.322314 1.409268

|

age_cat |

40 - 59 Years | 3.068526 .1387966 24.79 0.000 2.8082 3.352986

60 - 79 Years | 9.868963 .429211 52.64 0.000 9.062581 10.7471

80+ Years | 38.90732 1.767877 80.58 0.000 35.59216 42.53127

|

medcond_yn | 2.540803 .0834099 28.40 0.000 2.382471 2.709658

|

critical |

Critical | 6.569363 .1150923 107.45 0.000 6.347616 6.798858

|

county_size |

Micropolitan | .7098629 .0250794 -9.70 0.000 .6623714 .7607596

Rural/Noncore | .6763898 .0317228 -8.34 0.000 .6169864 .7415125

|

ses_svi |

25th to 50th percentile | .983919 .020984 -0.76 0.447 .9436388 1.025919

50th to 75th percentile | 1.735846 .0377696 25.35 0.000 1.663375 1.811474

>75th percentile | 1.658911 .0548996 15.29 0.000 1.554725 1.770079

|

_cons | .003299 .0005346 -35.26 0.000 .0024013 .0045324
