# Supplementary material for: Racial and neighborhood disparities in mortality among hospitalized COVID-19 patients in the United States: An analysis of the CDC case surveillance database
Source: PLOS Glob Public Health. 2022 Nov 16;2(11):e0000701. doi: 10.1371/journal.pgph.0000701 (PMC10022015; doi:10.1371/journal.pgph.0000701)
Supplement: S4 Table — (DOCX) [file pgph.0000701.s004.docx]

**Model including both race and neighborhood type**

------------------------------------------------------------------------------------------

death_yn | Odds ratio Std. err. z P>|z| [95% conf. interval]

-------------------------+----------------------------------------------------------------

racial_cat |

Black, Non-Hispanic | 1.029017 .022148 1.33 0.184 .9865108 1.073355

Hispanic/Latino | .9625102 .0231473 -1.59 0.112 .9181949 1.008964

Other races | .976235 .0308324 -0.76 0.446 .9176369 1.038575

|

minority_svi |

25th to 50th percentile | .9808063 .046929 -0.41 0.685 .8930083 1.077236

50th to 75th percentile | .9795259 .0429271 -0.47 0.637 .8989024 1.067381

>75th percentile | 2.084013 .0894595 17.11 0.000 1.915849 2.266937

|

sex | 1.386381 .0221105 20.48 0.000 1.343715 1.430401

|

age_cat |

40 - 59 Years | 2.939931 .1312229 24.16 0.000 2.693668 3.208709

60 - 79 Years | 9.037424 .3903598 50.97 0.000 8.303823 9.835834

80+ Years | 35.62515 1.618278 78.66 0.000 32.59048 38.9424

|

medcond_yn | 2.916497 .0947634 32.94 0.000 2.736555 3.108271

|

critical |

Critical | 5.932794 .1003435 105.27 0.000 5.739348 6.132759

|

county_size |

Micropolitan | .5786839 .0198338 -15.96 0.000 .5410872 .6188929

Rural/Noncore | .5116076 .0232524 -14.75 0.000 .4680047 .5592729

|

ses_svi |

25th to 50th percentile | .9678298 .020195 -1.57 0.117 .9290467 1.008232

50th to 75th percentile | 1.294772 .0264489 12.65 0.000 1.243957 1.347662

>75th percentile | 1.448566 .0449265 11.95 0.000 1.363134 1.539351

|

_cons | .0059757 .0004 -76.49 0.000 .0052409 .0068134
