# Supplementary material for: Racial and neighborhood disparities in mortality among hospitalized COVID-19 patients in the United States: An analysis of the CDC case surveillance database
Source: PLOS Glob Public Health. 2022 Nov 16;2(11):e0000701. doi: 10.1371/journal.pgph.0000701 (PMC10022015; doi:10.1371/journal.pgph.0000701)
Supplement: S5 Table — (DOCX) [file pgph.0000701.s005.docx]

**Distribution of neighborhoods in the west**

Neighbourhood type | Freq. Percent Cum.

------------------------+-----------------------------------

<=25th percentile | 5 0.05 0.05

25th to 50th percentile | 29 0.30 0.35

50th to 75th percentile | 607 6.30 6.65

>75th percentile | 8,995 93.35 100.00

------------------------+-----------------------------------

Total | 9,636 100.00
