# Supplementary material for: Racial and neighborhood disparities in mortality among hospitalized COVID-19 patients in the United States: An analysis of the CDC case surveillance database
Source: PLOS Glob Public Health. 2022 Nov 16;2(11):e0000701. doi: 10.1371/journal.pgph.0000701 (PMC10022015; doi:10.1371/journal.pgph.0000701)
Supplement: S6 Table — (DOCX) [file pgph.0000701.s006.docx]

**Table of Missing data**

**Total N= 528,769**

| **Variable** | **Missing**  **N (%)** | **Observed**  **N (%)** |
| --- | --- | --- |
| **Primary variables of interest** |  |  |
| Race | 130,254  (24.63) | 398,515  (75.37) |
| Sex | 6,026  (1.14) | 522,743  (98.86) |
| Death | 155,658  (29.44) | 373,111  (70.56) |
| Comorbidity | 327,326  (61.90) | 201,443  (38.10) |
| Age group | 2,529  (0.48) | 526,240  (99.52) |
| Socioeconomic status | 6,487  (1.23) | 522,282  (98.77) |
| County Size | 6,487  (1.23) | 522,282  (98.77) |
| Disease severity | 284,811  (53.86) | 243,958  (46.14) |
| Neighborhood type | 6,487  (1.23) | 522,282  (98.77) |
