# Supplementary material for: Racial and neighborhood disparities in mortality among hospitalized COVID-19 patients in the United States: An analysis of the CDC case surveillance database
Source: PLOS Glob Public Health. 2022 Nov 16;2(11):e0000701. doi: 10.1371/journal.pgph.0000701 (PMC10022015; doi:10.1371/journal.pgph.0000701)
Supplement: S7 Table — (DOCX) [file pgph.0000701.s007.docx]

**The Imputation Model for missing Data**

The CDC COVID-19 Case Surveillance Restricted Access database contained significant amounts of missing data (>5%) for the primary outcome (death), the primary predictor (race) and for the other covariates to be included in the model. The sample size before removing any missing data was 528,769. The Key differences in the outcome (death) between patients with complete and missing data for race, comorbidity and disease severity are shown in the table below.

**Missing Table A. Total N= 528,769**

| **Variable** | **Death** | | |
| --- | --- | --- | --- |
| **Race** | **No (%)** | **Yes (%)** | **Total (N)** |
| Complete | 67.53 | 32.47 | 283,724 |
| Missing | 79.29 | 20.71 | 89,393 |
| **Comorbidity** |  |  |  |
| Complete | 63.22 | 36.78 | 144,875 |
| Missing | 74.87 | 25.13 | 228,242 |
| **Disease Severity** |  |  |  |
| Complete | 70.93 | 29.07 | 188,545 |
| Missing | 69.76 | 30.24 | 184,572 |

We assumed the data were missing at random (MAR) and adjusted for missingness using multiple imputation. We imputed 20 datasets using the multiple imputation with chained equations (MICE) algorithm. We chose the MICE algorithm because our dependent and independent variables are binary/categorical.

Information on comorbidity and disease severity were not reported by several states resulting in significant amounts of missing data in these variables. The degree of missingness in the variables for race and death also varied significantly between states. Hence, to improve the prediction of these variables and maintain the plausibility of the missingness at random assumption, we included ‘state of residence’ as an auxiliary variable in our imputation model. The month of case report was also predictive of missingness in the outcome (death) and was included as auxiliary variable in the imputation model. The proportion of missing data for the primary variables of interest and the auxiliary variables are shown in the table below.

**Missing Table B**

| **Variable** | **Missing %** | **Observed %** |
| --- | --- | --- |
| **Primary variables of interest** |  |  |
| Race | 24.63 | 75.37 |
| Sex | 1.14 | 98.86 |
| Death | 29.44 | 70.56 |
| Comorbidity | 61.90 | 38.10 |
| Age group | 0.48 | 99.52 |
| Socioeconomic status | 1.23 | 98.77 |
| County Size | 1.23 | 98.77 |
| Disease severity | 53.86 | 46.14 |
| Neighborhood type | 1.23 | 98.77 |
| **Auxiliary Variables** |  |  |
| State of residence | 0% | 100% |
| month of case report | 0% | 100% |

**Post-Imputation**

Descriptive statistics for the demographic and clinical characteristics of hospitalized COVID-19 patients were conducted across the different racial groups using chi-square (Table S1).

To evaluate the primary objective, we ran separate univariate logistic regression models which included only the outcome of interest (death) and the primary predictors (race and neighborhood type). We also ran separate multivariable logistic regression models which included the outcome, primary predictors and other sociodemographic and health-related variables including age group, gender, socioeconomic status, presence of comorbidity, disease severity, and county size. These are shown in tables S2 and S3

For the regional analyses, we ran 4 different multivariable logistic regression models (one for each census region) which included the outcome of interest (death), the primary predictor (race) and other sociodemographic and health-related variables including age group, gender, socioeconomic status, presence of comorbidity, disease severity, and county size (table S4).

**Table A:** Demographic and clinical characteristics of hospitalized COVID-19 patients

| **Demographic and Clinical variables** | **Total**  **(%)** | **White, Non-Hispanic**  **(%)** | **Black, Non-Hispanic**  **(%)** | **Hispanic**  **(%)** | **Others**  **(%)** |
| --- | --- | --- | --- | --- | --- |
| **Gender** |  |  |  |  |  |
| Female | 47.50 | 46.90 | 51.79 | 44.75 | 47.00 |
| Male | 52.50 | 53.10 | 48.21 | 55.25 | 53.00 |
| **Age Group** |  |  |  |  |  |
| <40 years | 15.05 | 8.92 | 15.71 | 25.23 | 18.56 |
| 40-59 years | 27.55 | 19.67 | 31.12 | 38.41 | 31.07 |
| 60-79 years | 39.16 | 44.37 | 40.78 | 28.08 | 36.99 |
| 80+ years | 18.24 | 27.04 | 12.39 | 8.28 | 13.38 |
| **Comorbidities** |  |  |  |  |  |
| Absent | 14.52 | 14.32 | 9.46 | 18.62 | 17.36 |
| Present | 85.48 | 85.68 | 90.54 | 81.38 | 82.64 |
| **Disease Severity** |  |  |  |  |  |
| Non-critical | 70.40 | 73.05 | 69.57 | 67.92 | 65.55 |
| Critical | 29.60 | 26.95 | 30.43 | 32.08 | 34.45 |
| **Socioeconomic Status** |  |  |  |  |  |
| Quartile 1 | 23.36 | 30.89 | 13.13 | 18.16 | 23.37 |
| Quartile 2 | 31.34 | 32.93 | 26.92 | 34.13 | 27.21 |
| Quartile 3 | 32.53 | 26.50 | 40.70 | 35.28 | 36.00 |
| Quartile 4 | 12.77 | 9.68 | 19.25 | 12.43 | 13.42 |
| **Neighborhood Type** |  |  |  |  |  |
| Quartile 1 | 3.46 | 7.38 | 0.09 | 0.08 | 0.53 |
| Quartile 2 | 6.63 | 12.29 | 2.84 | 0.99 | 1.78 |
| Quartile 3 | 17.83 | 25.15 | 17.33 | 7.81 | 7.88 |
| Quartile 4 | 72.08 | 55.18 | 79.74 | 91.13 | 89.80 |
| **Census Region** |  |  |  |  |  |
| Northeast | 32.77 | 27.68 | 33.51 | 38.57 | 31.57 |
| Midwest | 21.89 | 27.88 | 20.31 | 14.89 | 13.58 |
| South | 23.84 | 24.64 | 32.94 | 18.90 | 10.82 |
| West | 21.50 | 19.80 | 13.24 | 27.64 | 34.03 |
| **County Size** |  |  |  |  |  |
| Metropolitan | 89.09 | 83.35 | 91.51 | 95.99 | 94.52 |
| Micropolitan | 6.32 | 9.56 | 4.38 | 2.97 | 3.27 |
| Rural/Noncore | 4.59 | 7.09 | 4.11 | 1.04 | 2.21 |
| **Death** |  |  |  |  |  |
| No | 71.11 | 68.68 | 70.78 | 75.99 | 71.83 |
| Yes | 28.89 | 31.32 | 29.22 | 24.01 | 28.17 |

**Table B:** Logistic regression results for racial disparities in mortality among hospitalized COVID-19 patients

| **Model 1 (reference is White)** | **Unadjusted OR**  **(95% CI)** | **Adjusted OR,**  **(95% CI^*^)** |
| --- | --- | --- |
| Black, Non-Hispanic | 0.91 (0.89, 0.92) | 1.13 (1.11, 1.16) |
| Hispanic/Latino | 0.69 (0.68, 0.71) | 1.14 (1.10, 1.17) |
| Other races | 0.86 (0.84, 0.88) | 1.06 (1.02, 1.10) |
| **Model 2 (reference is low vulnerability)** |  |  |
| Quartile 2 | 1.05 (1.10, 1.10) | 1.01 (0.95, 1.07) |
| Quartile 3 | 1.06 (1.02, 1.11) | 1.09 (1.03, 1.15) |
| Quartile 4 | 1.37 (1.32, 1.42) | 1.42(1.34, 1.50) |

^*^Adjusted for age group, gender, presence of comorbidity, disease severity, socioeconomic status, and county size.

**Table C:** Results of the fully adjusted multivariate logistic regression model

| **Variables** | **Model 1 Adjusted OR, 95% CI^*^** | **Model 2 Adjusted OR, 95% CI^*^** |
| --- | --- | --- |
| **Racial Group (Reference category is White, non-Hispanic)** |  |  |
| Black, Non-Hispanic | 1.13 (1.11, 1.16) | - |
| Hispanic/Latino | 1.14 (1.10, 1.17) | - |
| Other races | 1.06 (1.02, 1.10) | - |
| **Neighborhood type (reference is low vulnerability)** |  |  |
| Quartile 2 (moderate vulnerability) | - | 1.01 (0.95, 1.07) |
| Quartile 3 (high vulnerability) | - | 1.09 (1.03, 1.15) |
| Quartile 4 (very high vulnerability) | - | 1.42(1.34, 1.50) |
| **Sex (Reference category is Female)** |  |  |
| Male | 1.34 (1.31, 1.36) | 1.34 (1.31, 1.36) |
| **Age Group (Reference category is <40 years)** |  |  |
| 40-59 years | 3.00 (2.84, 3.17) | 3.01 (2.85, 3.18) |
| 60-79 years | 10.29 (9.76, 10.85) | 10.27 (9.73, 10.83) |
| 80+ years | 38.01 (35.94, 40.20) | 37.41 (35.35, 39.58) |
| **Presence of comorbidities (Reference category is Absent)** |  |  |
| Present | 2.84 (2.68, 3.01) | 2.81 (2.65, 2.98) |
| **Disease severity (Reference category is non-critical)** |  |  |
| Critical | 6.08 (5.91, 6.25) | 6.08 (5.91, 6.26) |
| **County size (Reference category is Metropolitan)** |  |  |
| Micropolitan | 0.57 (0.55, 0.60) | 0.66 (0.64, 0.69) |
| Rural/Noncore | 0.61 (0.59, 0.64) | 0.72 (0.99, 0.75) |
| **Socioeconomic status (Reference category is Quartile 1)** |  |  |
| Quartile 2 | 1.08 (1.05, 1.10) | 1.04 (1.01, 1.07) |
| Quartile 3 | 1.42 (1.39, 1.46) | 1.36 (1.32, 1.39) |
| Quartile 4 | 1.50 (1.45, 1.55) | 1.43 (1.38, 1.48) |

^*^Adjusted for age group, gender, presence of comorbidity, disease severity, socioeconomic status, and county size.

**Table D:** Comparing racial disparities in mortality among hospitalized COVID-19 patients across the 4 census regions

| **Racial Group (White is reference category)** | **Northeast OR (95% CI)** | **Midwest OR (95% CI)** | **South OR, (95% CI)** | **West, OR**  **(95% CI)** |
| --- | --- | --- | --- | --- |
| Black, Non-Hispanic | 1.04 (0.99, 1.08) | 1.16 (1.11, 1.22) | 1.02 (0.98, 1.06) | 0.90 (0.83, 0.96) |
| Hispanic/Latino | 1.02 (0.98, 1.07) | 1.31 (1.23, 1.40) | 0.81 (0.77, 0.86) | 1.09 (1.03, 1.16) |
| Other races | 0.97 (0.93, 1.02) | 0.90 (0.82, 0.99) | 0.83 (0.75, 0.93) | 1.04 (0.97, 1.12) |
| **Neighborhood type (reference is low vulnerability)** |  |  |  |  |
| Quartile 2 | 1.38 (1.17, 1.62) | 0.98 (0.90, 1.07) | 0.96 (0.86, 1.07) | 1.46 (1.07, 2.00) |
| Quartile 3 | 1.88 (1.63, 2.18) | 0.97 (0.90, 1.05) | 0.97 (0.88, 1.07) | 2.03 (1.52, 2.72) |
| Quartile 4 | 2.24 (1.94, 2.58) | 1.69 (1.56, 1.83) | 0.93 (0.85, 1.03) | 1.90 (1.41, 2.56) |

^*^Adjusted for age group, gender, presence of comorbidity, disease severity, socioeconomic status, and county size.
