# Supplementary material for: Racial and neighborhood disparities in mortality among hospitalized COVID-19 patients in the United States: An analysis of the CDC case surveillance database
Source: PLOS Glob Public Health. 2022 Nov 16;2(11):e0000701. doi: 10.1371/journal.pgph.0000701 (PMC10022015; doi:10.1371/journal.pgph.0000701)
Supplement: S1 Data — (DOCX) [file pgph.0000701.s008.docx]

| Datasets used in the Study | Links | Contact information for dataset owner |
| --- | --- | --- |
| CDC COVID-19 Case Surveillance Restricted Access Detailed Data | <https://data.cdc.gov/Case-Surveillance/COVID-19-Case-Surveillance-Restricted-Access-Detai/mbd7-r32t/> | [eocevent394@cdc.gov](mailto:eocevent394@cdc.gov) |
| CDC NCHS Urban-Rural Classification Scheme for Counties | <https://www.cdc.gov/nchs/data_access/urban_rural.htm#Data_Files_and_Documentation> | Publicly available |
| CDC Agency for Toxic Substances and Disease Registry, Social Vulnerability Index | <https://www.atsdr.cdc.gov/placeandhealth/svi/data_documentation_download.html> | Publicly available |
